# Magnetic Resonance Imaging Reveals Novel Insights into the Dual Mode of Action of Bisacodyl: A Randomized, Placebo‐controlled Trial in Constipation

**DOI:** 10.1002/cpt.3532

**Published:** 2024-12-16

**Authors:** Abdulsalam Aliyu, Neele Dellschaft, Caroline Hoad, Hannah Williams, Emily Gaudoin, Sarah Sulaiman, Colin Crooks, Penny Gowland, Alexia Aran, Robert Lange, Beatrice Bois De Fer, Maura Corsetti, Luca Marciani, Robin Spiller

**Affiliations:** ^1^ Nottingham NIHR Research Centre University of Nottingham Nottingham UK; ^2^ School of Medicine University of Nottingham Nottingham UK; ^3^ Sir Peter Mansfield Imaging Centre University of Nottingham Nottingham UK; ^4^ Sanofi Barcelona Spain; ^5^ Sanofi Frankfurt am Main Germany; ^6^ Sanofi Neuilly‐sur‐Seine France

## Abstract

Bisacodyl is a widely used laxative that stimulates both motility and secretion. Our aim was to exploit the unique capabilities of MRI to define bisacodyl's mode of action. Two placebo‐controlled cross‐over trials were performed, one using a single dose of Bisacodyl 5 mg while the second dosed daily for 3 consecutive days. Serial MRI was performed every 75 minutes. Primary endpoint: ascending colon water content as assessed by T1AC AUC_300–450 minutes_. Secondary endpoints included: small bowel water content, whole gut transit time (WGTT), colonic volumes, stool frequency, and consistency using Bristol Stool Form Score (BSFS). Exploratory endpoints: changes in the serial segmental volumes were quantified from the number of “mass movements” defined as episodes when segmental volume change from the previous scan was > 20% of baseline volume. We also measure the time to defecate after dosing. After 3 days of bisacodyl, ascending colon water content (T1) was 62% greater than after placebo, mean difference T1 AUC_300–450 minutes_ 50.2 (61.0) sec.min, 95% CI (9.2, 91.2), *P* = 0.02, while after a single dose difference was only 11% (*P* = 0.58). Both single and repeated doses shortened WGTT (*P* < 0.049) and time to defecate (*P* 0.01). Only repeated doses significantly increased small bowel water content (*P* < 0.03), the number of “mass movements” (*P* = 0.048), bowel frequency (*P* = 0.006), and BSFS (*P* = 0.03). Repeated, compared to single dosing of Bisacodyl, additionally increases small bowel and colon water content and increases the number of “mass movements” thereby increasing its laxative effect. MRI is a non‐invasive, patient‐acceptable technique for evaluating drugs which alter secretion and/or motility.

**Study Highlights**

**WHAT IS THE CURRENT KNOWLEDGE ON THE TOPIC?**

Bisacodyl is an effective laxative that stimulates motility and secretion but no previous techniques have been able to simultaneously assess both secretion and motility in humans, nor its impact on the gut water content.

**WHAT QUESTION DID THIS STUDY ADDRESS?**

Our primary endpoint was the effect of bisacodyl on the water content of the ascending colon using the MRI parameter T1, a novel way of non‐invasively assessing luminal water content. We also wanted to know if repeated dosing was more effective than a single dose and why.

**WHAT DOES THIS STUDY ADD TO OUR KNOWLEDGE?**

When compared to a placebo a single dose of bisacodyl has a predominantly prokinetic effect, accelerating whole gut transit and reducing the time from dose ingestion to defecation without altering small or large bowel water content or stool consistency. In contrast, three repeated daily doses increase both small bowel water content and the wateriness of colonic contents as assessed by T1. They also stimulated mass movements which accelerated gut transit, softened stool, and reduced time to defecation.

**HOW MIGHT THIS CHANGE CLINICAL PHARMACOLOGY OR TRANSLATIONAL SCIENCE?**

Patients who fail to respond to a single dose of 5 mg bisacodyl should be encouraged to take repeated daily doses which may well soften stool and relieve constipation. MRI can evaluate in detail the mode of action of drugs that alter both intestinal secretions and/or motility with a relatively small Number Needed To Test.

Bisacodyl is a stimulant laxative, widely used to treat constipation for many years, and shown to be among the most effective laxatives.^1^ Bisacodyl 10 mg induces high‐amplitude contractions (HAPCs) when administered intra‐colonically and after oral administration, 5 mg bisacodyl stimulates ascending colon transit^2^. Previous animal^3^ and human studies using invasive perfusion techniques and ileostomy patients, have demonstrated that bisacodyl stimulates secretion and motility^4^, ^5^, ^6^ however, until recently it was not possible to study simultaneously motility and secretion in the undisturbed gut in humans. MRI can non‐invasively measure intestinal motility, gut volumes, and water content enabling evaluation of both secretory and prokinetic effects in a single study.^7^ The aim of the studies reported here was to use MRI to define the mechanisms underlying the laxative effect of bisacodyl a treatment which is obtained over the counter and used by patients both intermittently as required as well as more regularly. Specifically, our aim was to define the role of prokinetic and prosecretory effects of both a single dose of 5 mg bisacodyl and 5 mg given daily for 3 days to determine whether giving repeated doses alters the mode of action. The 5 mg dose was selected for the current studies since in a confirmatory clinical trial conducted with a 10 mg starting dose, many patients reduced the daily dosage to 5 mg during the first week, suggesting 5 mg is effective.^8^

## METHODS

We enrolled adult subjects meeting Rome IV criteria for Functional Constipation and self‐medicating with occasional laxatives no more than four times in the month before eligibility confirmation. Two independent studies were performed. The first study (Single dose) was designed to examine the effect of a single dose of bisacodyl to mimic its use in constipated subjects who took laxatives occasionally. The second study (Repeated dose) examined the effect of 5 mg bisacodyl administered once daily for 3 consecutive days.

### Participants

Participants were recruited from public noticeboards on Nottingham University campuses and adverts on social media. Full inclusion and exclusion criteria are provided in **Supplement** **1.1** **&** **1.2**.

### Study design

**Figure 1:**
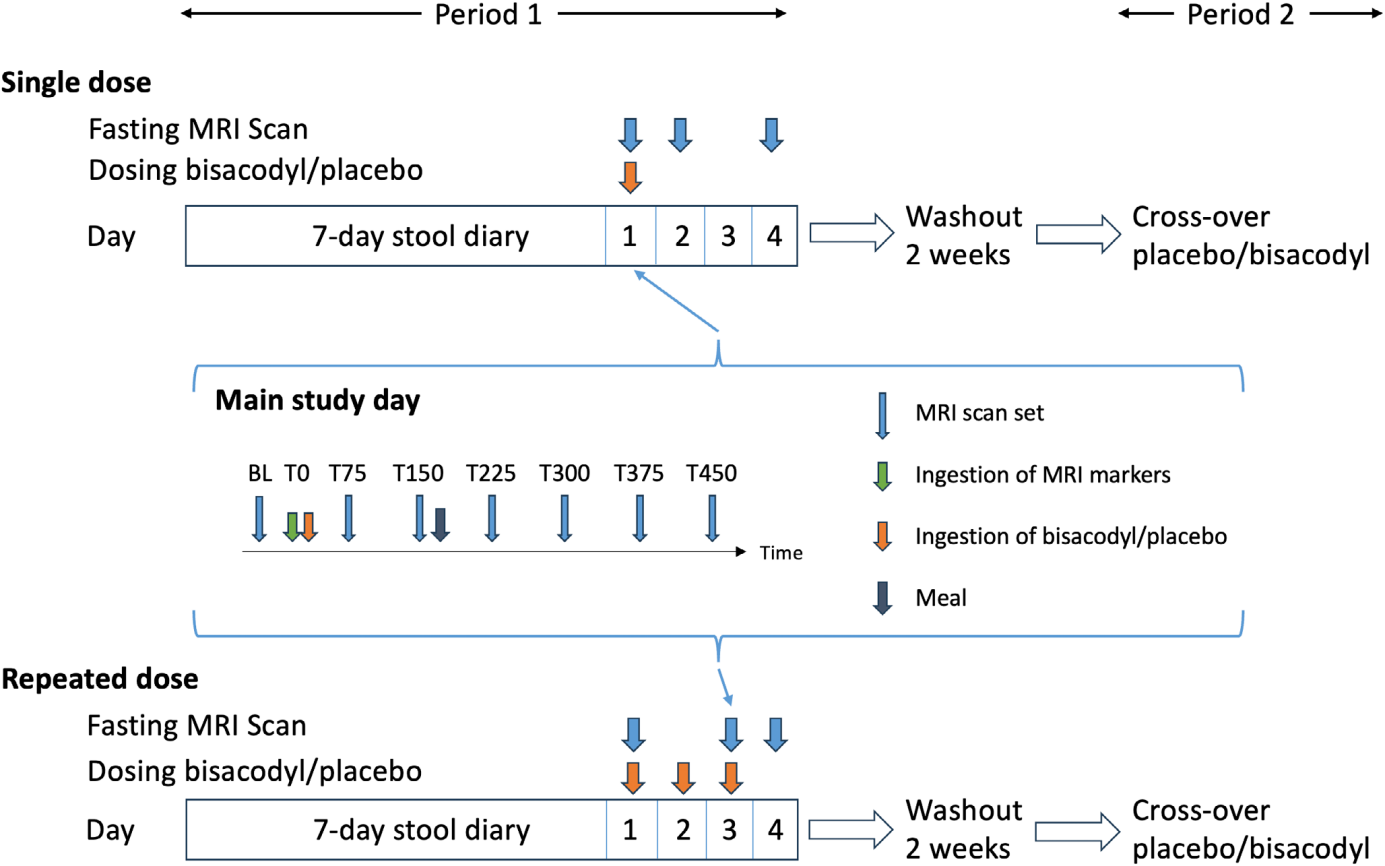
Summary of study intervention for the two periods of the studies (Single and repeated dose). Both studies involved a preliminary stool diary to confirm eligibility followed by enrolment and randomization to either bisacodyl/placebo or placebo/bisacodyl. A further 7‐day stool diary was completed prior to starting period 2 during the 2 weeks washout period to confirm ongoing eligibility. The main study day was identical for both studies.

These were randomized, investigator and patient‐blinded, placebo‐controlled, cross‐over studies with 2 weeks wash out periods after which the ongoing presence of constipation was confirmed before proceeding as shown in **Figure** **1**. This was done by asking the participants to complete a 7‐day screening symptom diary which required that they recorded at least 2 days in the 7‐day diary with either hard stools (Bristol Stool form Score, BSFS, 1 or 2) or no bowel movement (BM).

All participants underwent serial MRI scanning at the Sir Peter Mansfield Imaging Centre as detailed below.

#### Single dose

The main study day with serial MRI scanning took place on Day 1. Patients arrived after an overnight (approximately 14 hours) fast and underwent serial scanning (every 75 minutes) both at baseline and for 6 hours after ingestion of study drug as well as follow‐up scans 24 and 72 hours later (**Figure** **1**).

#### Repeated dose

This was similar to the single dose study but the main study day with serial MRI scanning took place on Day 3. Subjects attended for a fasting scan before the first drug intake on Day 1, the second drug intake occurring at home with the third dose as part of the main study day on Day 3. There was a single follow‐up fasting scan 24 hours later (**Figure** **1**). A standard 331 kcal rice pudding and orange juice meal, as previously used,^9^ was taken after the 150 minutes scan for both studies. All subjects were asked to avoid certain foods likely to increase colonic gas as detailed in **Supplement** **1.3**.

### Randomization and blinding

Participants were assigned a unique randomization number which determined the allocation to one of the two treatment sequences according to a code generated by the statistics department at Sanofi (Further details **Supplement** **1.4–1.6**). No research staff knew which treatment was given.

### Endpoints

Primary endpoint: This was the difference in water content in the ascending colon between placebo and bisacodyl measured indirectly by T1 signal intensity. T1 is a time constant that reflects the speed with which protons in water return to their unexcited state after absorbing a pulse of electromagnetic radiation. The T1 of water is 3–4 seconds while the T1 of the descending colon in health is around 0.56, a value which correlated well with the water content of the subsequent stool passed^10^ so it can be regarded as an approximate measure of colonic water. We used T1 of the ascending colon as our primary endpoint as a measure of chyme water content since this is where increased small bowel secretions would have their biggest impact and the ascending colon provides an easily imaged area with adequate content to allow the T1 measurement (for more details see **Supplement** **1.7.1**). We used T1 AUC_300–450 minutes_ after the last study drug intake, Day 1 for the single dose study, and Day 3 for the repeated dose study since previous studies indicate that the maximum motility response occurs between 5 and 6 hours after oral bisacodyl intake.^11^ The units of the T1 AUC_300–450 minutes_ is seconds.minutes (sec.min).

Secondary endpoints included (1) the difference in placebo versus bisacodyl treatment of ascending and descending colon water content as assessed by T1 (sec) 24 hours post‐dosing, (2) small bowel water content (mL) and (3) motility (arbitrary units), (4) ascending and descending colonic motility (arbitrary units), (5) ascending, transverse, descending and rectosigmoid colonic volumes (mL) from 0 to 450 minutes post‐dosing, (6) whole gut transit in hours assessed by MRI marker position at 24 hours, and (7) frequency and consistency of stool as assessed by Bristol Stool Form Scale.

There were no changes to the primary or secondary endpoints, however, before the code break (unblinding) we included some additional exploratory endpoints. While individual segmental volumes mostly changed little from scan to scan, there were occasional substantial changes. These were similar to the changes in colon volume induced by a meal observed in previous barostat studies^12^ attributed to variation in colon tone and in a study using cinefluorography to examine the response to a laxative similar to bisacodyl namely oxyphenacetin, previously described as “mass movements”.^13^ We calculated the number of time periods when sequential scans showed a change of > 20% of baseline volume using the same time frame (300–450 minutes post‐dosing) as for the primary endpoint when bisacodyl would be expected to act. We hereafter refer to these events as “mass movements”. The 20% cut‐off was chosen based on previous scintigraphic studies demonstrating that such large volume changes were frequent in lactulose‐induced diarrhea and significantly reduced by Mebeverine.^14^ The value is similar to the mean change observed in the transverse colon (24%) using the barostat after a 1,000 kcal meal,^12^ a stimulus known to stimulate colonic motility.

Participants completed a daily stool diary throughout the study, documenting Bristol Stool Form score for each bowel movement. This allowed analysis of the time to first defecation after drug intake, a measure relevant to the patient's perception of efficacy.

### MRI scanning details and analysis

We measured colon water content T1, small bowel water content and motility, colon volume and motility as previously described^10^, ^15^, ^16^, ^17^, ^18^ for each of the scans acquired every 75 minutes for 450 minutes on the full study days as well as for scans acquired at baseline, 24 hours, and 72 hours in fasting state. Full details and references are provided in **Supplement** **1.7**.

Previous studies had validated T1 as a measure of chyme water content since T1 of the distal colon chyme correlates with the water content of the next stool passed^10^ (for more details see **Supplement** **1.7.1**). Ascending colon T1 has been shown to be sensitive to the water trapping induced by both psyllium and kiwifruit,^10^, ^18^ treatments with known laxative effect.

Small bowel water content was assessed from a single shot, fast spin echo sequence, previously validated against the instillation of known volumes of saline.^15^ Small bowel motility was assessed using a cine MRI bTFE acquisition.^19^

Colonic images were analyzed by manually outlining the colon to create regions of interest to calculate colon volumes from images acquired every 75 minutes and were also used to assess position of the transit pills and calculate transit as previously described.^20^ Colonic volumes have been shown to be reproducible with a coefficient of variation day to day of 14%.^21^ Colonic Motility index was assessed from colonic wall movement using a 2‐slice cine MRI as previously described.^21^

### Statistical analysis

The sample size calculation was performed based on the primary endpoint, ascending colon water content as assessed by T1 AUC_300–450 minutes_. In a previous study in chronic constipation, treatment with psyllium showed an increase in ascending colon T1 AUC_300–420 minutes_ versus placebo of mean (Standard Deviation SD) 45.8 (36.3) sec.min (unpublished data from Major et al.^10^), a difference we considered to represent the minimum clinically important treatment difference.

We calculated that a sample size of 10 participants (5 per sequence group) would allow detection of a difference between bisacodyl and placebo of 45.8 seconds for the primary endpoint of T1 ascending colon AUC_300–450 minutes_, with a within‐subject SD of 36.3 sec.min using a two‐sided paired *t*‐test at a nominal alpha level of 5% and a power of 90%.

Allowing for dropout or technical failure we aimed to randomize up to 18 subjects (9 per sequence group) to ensure at least 10 evaluable participants in each cross‐over study. We used a modified Intention to Treat analysis including those randomized with an evaluable primary endpoint for the two treatment periods, that is, technically satisfactory MRI scans.

Continuous data are presented as mean (SD) or median (IQR) if non‐normally distributed. To formally test for an association of the primary endpoint and secondary endpoints with treatment, a paired *t*‐test was used. A *P*‐value of < 0.05 was considered significant. Differences in stool consistency and number of time epochs with > 20% volume changes were assessed using a Wilcoxon signed rank test. A linear mixed‐effect model for repeated measures was used to assess the effect of time on T1.

Statistical analysis was performed using Excel, R (Version 4.2.2) and Prism Graph Pad (Version 9.4.).

The studies were conducted from September 25, 2020 to July 22,2022 in accordance with the Declaration of Helsinki,^22^ approved by the University of Nottingham Medical School Ethics Committee (Reference No: 420‐1911 and 419‐1911) and preregistered on ClinicalTrials.gov (Identifier: NCT04132661 and NCT04129788). All authors had access to the study data and reviewed and approved the final manuscript.

## RESULTS

### Demographics

Participants in both the single and repeated dose studies were predominantly middle‐aged women, (age, mean (SD) 50.0 and 53.5 (16.3) years gender, M/F 2/9 and 0/11, respectively). Fourteen were randomized to the single dose and 15 to the repeated dose study (Full details of recruitment, demographics and Consort diagram see **Supplement** **1.3–1.5**). Six participants were enrolled in both studies.

### Adverse events

The treatments were well tolerated with only minor symptoms like cramps, gas, and bloating.

### Primary endpoint

**Figure 2:**
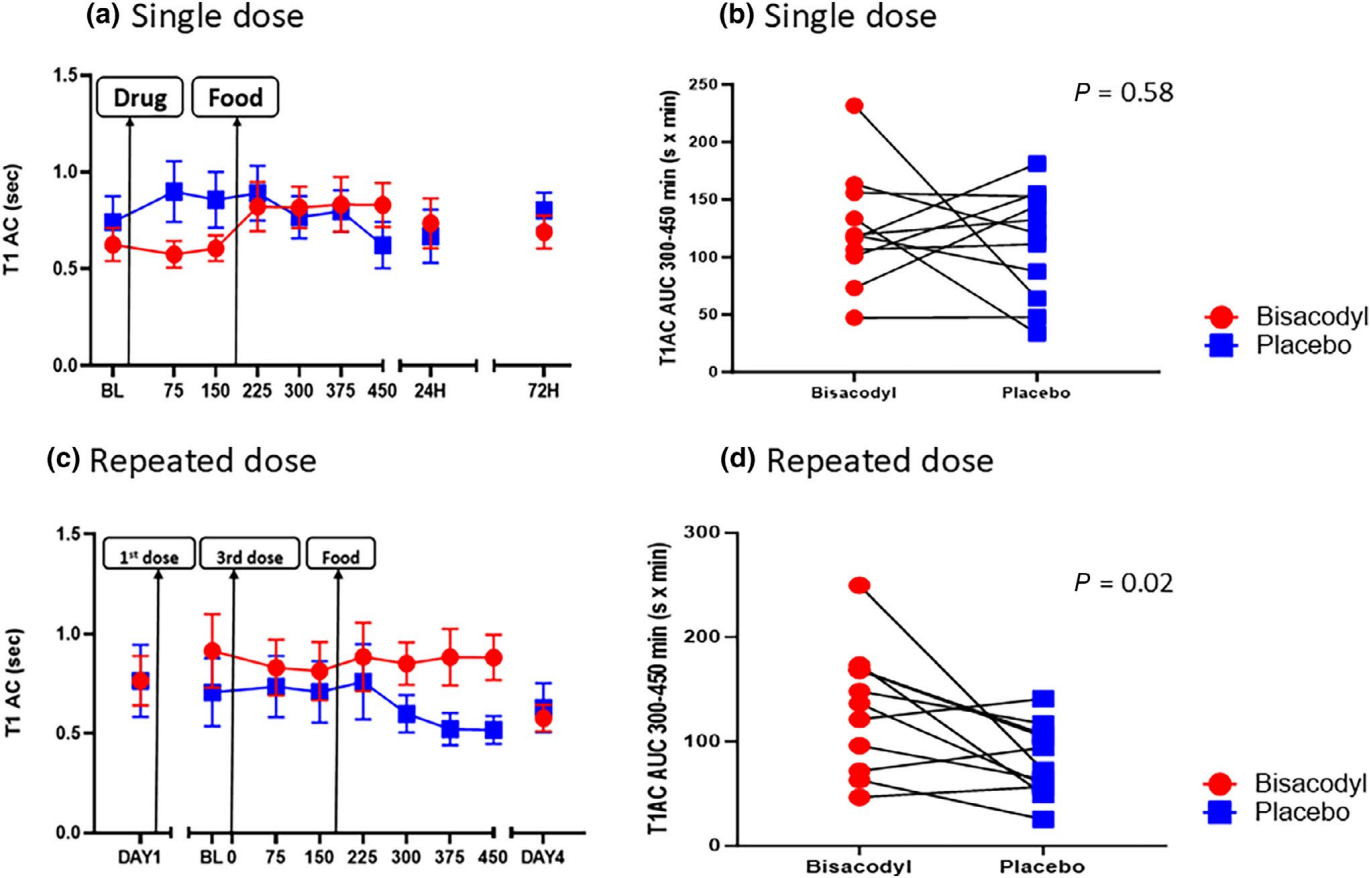
T1 ascending colon before and after intake of study drug. Panel **a**. Time course of T1 ascending colon (T1AC) following a single dose (mean (SD), *n* = 11). A linear mixed‐effect analysis of the variance model for repeated measures showed T1 on bisacodyl increased significantly being 0.60 during 0–150 minutes and 0.83 during 150–450 minutes, *P* = 0.0009 while there was no significant change on placebo (*P* = 0.102). Panel **b** Individual data points for T1 ascending colon AUC_300–450 minutes_. There was no significant difference between bisacodyl and placebo, *P* = 0.58. Panel **c** Time course of T1 ascending colon (T1AC) shown as mean (SD) *n* = 11. First dose given on Day 1, second dose at home on Day 2, and third dose on Day 3 followed by serial scanning. T1 on placebo fell from an average of 0.72 seconds for 0–150 minutes to 0.55 second for 300–450 minutes (*P* = 0.013) however on bisacodyl no change was observed (*P* = 0.82). Panel **d** Individual data points for T1 ascending colon AUC_300–450 minutes_. There was a significant increase in T1 ascending colon AUC_300–450 minutes_ after repeated doses of bisacodyl which was 131.2 (59.6) versus 81.0 (34.4) sec.min on placebo, *P* = 0.02.

After a single dose of bisacodyl, the water content of the ascending colon as assessed by T1AC AUC_300–450 minutes_ did not differ compared to placebo (*P* = 0.58). However, after 3 consecutive doses T1AC AUC_300–450 minutes_ was 62% greater on bisacodyl compared to placebo, mean difference being 50.2 (61.0) sec.min, 95% CI (9.2, 91.2, *P* = 0.02; **Figure** **2**). There was no period effect (*P* = 0.66 and 0.22) for the single and repeated dose study, respectively.

### Secondary endpoints

#### Ascending and descending water content T1 at 24 hours post‐dosing

These had returned to similar values as at baseline by 24 hours and 72 hours after a single dose and by 24 hours after repeated doses of bisacodyl (there was no 72‐hour scan in the repeated dose study). All values showed no difference between bisacodyl and placebo (**Figure** **2** **a,c**).

#### Small bowel water content

**Figure 3:**
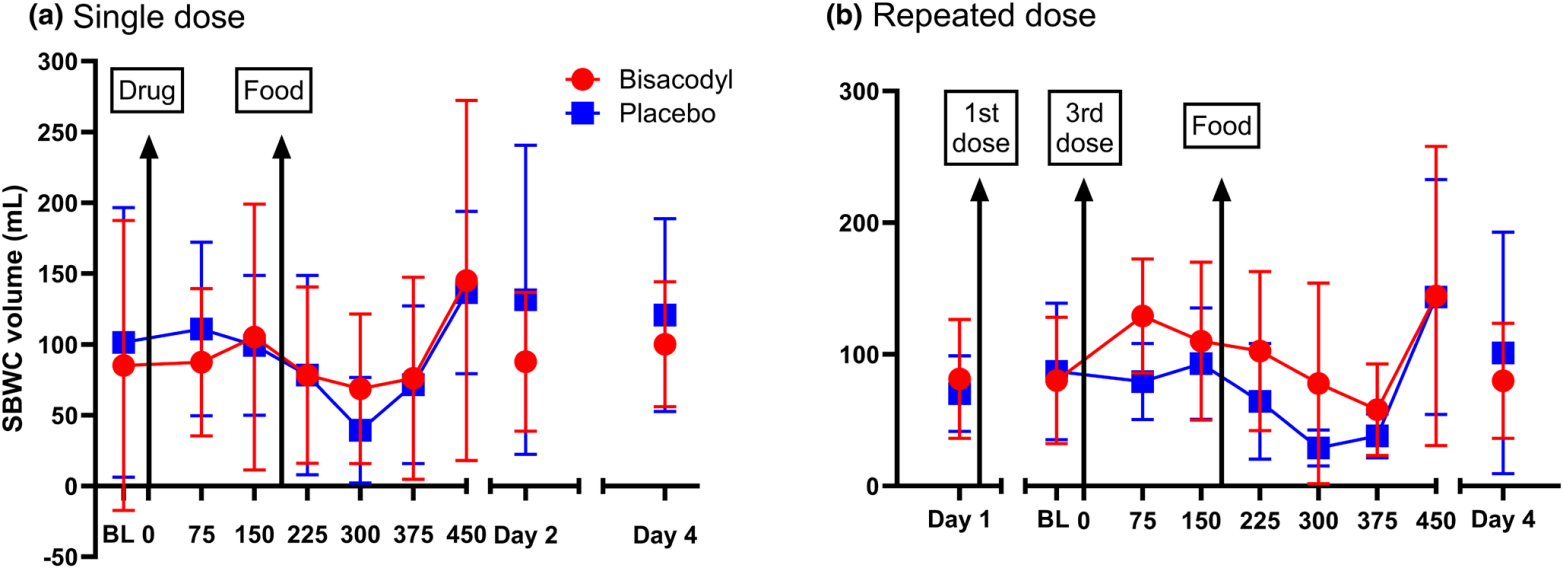
Small bowel water content changes over time (mean (SD)). Panel **a** Small bowel water content over time in the single dose study (mean (SD), *n* = 11) showing no difference between treatments. Panel **b** Data from repeated dose study showing small bowel water content was significantly greater on bisacodyl for both 0–150 and 150–450 minutes, (*n* = 11, *P* = 0.027 and 0.018, respectively).

The small bowel water content time profile showed its previously described pattern after the orange juice and rice pudding meal^9^ with a decrease followed by a rise (**Figure** **3**). After a single dose of bisacodyl there was no difference between bisacodyl and placebo for the 0–150 and 150–450 minute periods *P* = 0.22 and 0.29, respectively. However, in the repeated dose study both AUC_0–150 minutes_ and AUC_150–450 minutes_ showed significant mean differences between bisacodyl and placebo of 4,111 (5245) mL.min and 8,622 (10,105) mL.min, *P* = 0.027 and 0.018, respectively.

#### Small bowel and colon motility

Small bowel motility rose significantly (*P* < 0.005) promptly after drug ingestion and remained elevated throughout the study with no differences between bisacodyl or placebo in either single dose or repeated dose study (**Supplement** **1.7.3** **Figure** **S3**).

Ascending and descending colon motility measured as colon wall phasic movements were limited by technical difficulties related to the fact that the colon was less full and homogeneous in this study as compared to previous studies with Movicol, making it difficult to distinguish genuine colonic wall movement from respiratory artifact (detailed in **Supplement** **1.7.2**). This led to much reduced n values and no consistent treatment effect (data not shown).

#### Colonic volumes

Ascending, transverse and descending colon volumes as assessed by AUC_150–450mins_ did not alter after a single dose of bisacodyl however, the rectosigmoid colon did show a significant increase after bisacodyl as compared with placebo (mean difference (95% CI) 13,166 (1,568, 24,763), *P* = 0.03), an 18% increase consistent with a distal distension.

In contrast, after three repeated doses, there was a significant fall in ascending colon volume from 150 to 450 min with bisacodyl compared to placebo (AUC_150–450 minutes_ 13,445 (25,064 to 1826) mL, *P* = 0.03), a 17% decrease consistent with proximal contraction. The transverse, descending and rectosigmoid colon volumes showed no significant change in volume over time, nor any difference between bisacodyl and placebo (**Supplement Figure** **S4–S7**).

#### Whole gut transit time

Both single and repeated doses of bisacodyl accelerated the whole gut transit time of the MRI markers which fell from 63 (49.5, 94.5) hours on placebo to 22 (13, 61) hours on bisacodyl after a single dose (*n* = 11, *P* < 0.001) and from 57.4 (26.0, 83.7) to 15.6 (3.7, 57.4) hours after 3 repeated doses (*n* = 11, *P* = 0.049).

#### Numbers of “mass movements”

**Figure 4:**
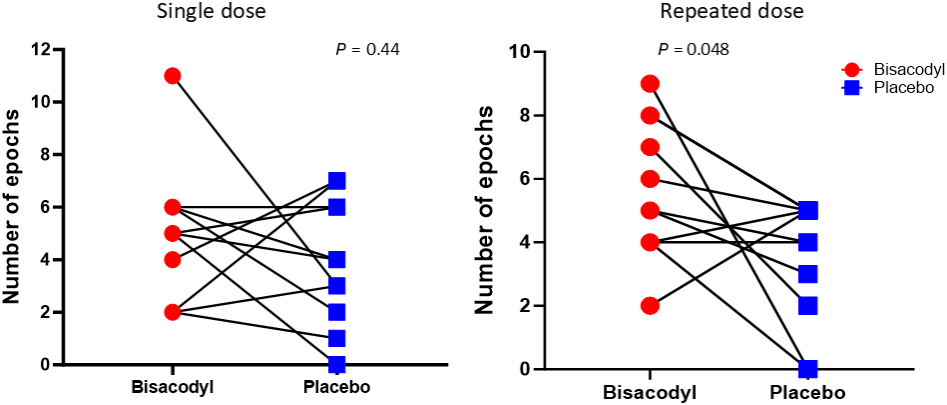
Number of epochs when segmental colon volume change was > 20% of baseline volume from 300 to 450 minutes (median (range)). Panel **a** after a single dose and Panel **b** following the third dose in the repeated dose study (*n* = 11 for both). Difference from placebo in single dose study *P* = 0.44, repeated doses study *P* = 0.048.

**Figure 5:**
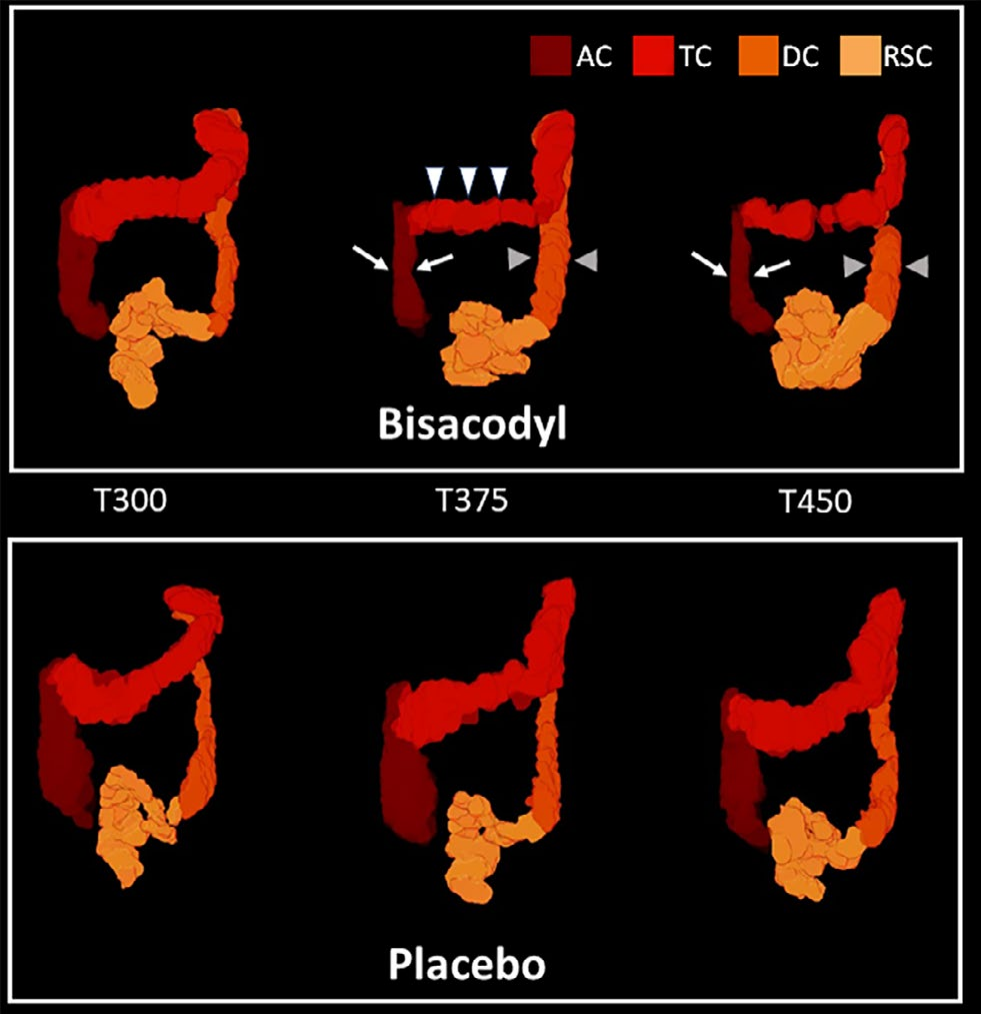
3D volume renderings of the MRI data, from a single participant in the repeated dose study. This shows the substantial changes in colon volumes after the third dose of bisacodyl when compared to placebo. The image at T 375 minutes shows the ascending colon volume reducing (contracting—white arrows) with simultaneous contraction of the transverse colon (white arrowheads). At the same time, the descending colon shows an increase in volume (relaxation/reduction in colon tone—light gray arrowheads). The rectosigmoid colon also shows an increase in volume (relaxation/reduction in colon tone) by 450 minutes. The volumes of the segments show little change over the same timeperiod in the placebo arm.

There were significantly more timeperiods (epochs) when the segmental volume change from the previous scan exceeded 20% of baseline in the repeated dose study, being 5.6 (2.2) on bisacodyl compared to 3.5 (2.0) on placebo, an increase of 60% (*P* = 0.048). The changes after a single dose were not significant (*P* = 0.44) (**Figure** **4**). The changes in ascending and transverse colon segmental volume seen during one such event are shown in **Figure** **5**. These are likely to reflect an increase in colon tone previously described to lead to “mass movement”.^13^ The Video (**Supplement** **1.10**) illustrates one mass movement recorded in a participant after bisacodyl intake in the single‐dose study.

#### Stool frequency, consistency and time to defecation

There was an increase in the number of bowel movements in the repeated dose study on Day 3 after the third dose of bisacodyl which rose from 0.5 (0.5) on placebo to 1.1 (0.5) on bisacodyl (*P* = 0.006). However, in the single‐dose study, the difference in bowel frequency on Day 1 was not significant being 0.6 (0.7) and 1.1 (0.55), respectively, *P* = 0.38.

Although not all participants opened their bowels on each day, for those that did, there was a softening of stool as shown by an increase in average BSFS on Day 3 in the repeated dose study which rose from 2.2 (1.3) on placebo to 4.9 (1.6) on bisacodyl reflecting a softening of stool (*P* = 0.015) while in the single‐dose study, the change from 2.4 (1.1) to 3.3 (1.7) on Day 1 was not significant (*P* = 0.18).

A single dose of bisacodyl significantly reduced time to defecate following dosing in the single‐dose study which fell from 24.5 (9.3, 38.8) hours on placebo to 10.5 (6.25, 16.5) hours on bisacodyl (*P* = 0.05), excluding the one patient who did not record a bowel movement on placebo before the study ended at 9:00 a.m. on Day 4. Including this patient by censoring at 72 hours in a log‐rank test showed no significant difference in time to defecation (*P* = 0.2). The repeated dose study showed reduced time to defecate after the third drug ingestion on Day 3, median time being 6.3 (3.6, 7.4) hours on bisacodyl compared to 12.5 (2.8, 22.3) hours on placebo, however, this was not significant (*P* = 0.96). As with the single‐dose study not all participants in the repeated dose study opened their bowels on each day but treating time to defecate > 24 hours as censored, so each day is a separate timeperiod, and using the Cox proportional hazards model shows the effect of treatment, significantly increased the chance of opening bowels by 72% across the 3 days (hazard ratio 1.72, 95% CI 1.14 to 2.60, *P* = 0.01).

## DISCUSSION

Our studies are the first to simultaneously investigate the effect of a stimulant laxative on both gut secretion and motility using MRI. While after a single dose there were no differences between bisacodyl and placebo for our primary endpoint T1AC AUC_300–450 minutes_, after three daily doses of bisacodyl there was a significant increase. We chose 3 days of usage to replicate the real world experience of bisacodyl usage in which most patients took 5 mg intermittently, averaging approximately 2 days per week over a 12 month period.^23^ After a single dose of bisacodyl, ascending colon water content increased significantly over 6 hours, a change which was not seen with placebo. In contrast after 3 doses, baseline ascending water content was already elevated and remained so during the study day (see **Figure** **2**). As observed in other MRI studies both in health and constipation,^10^ we found T1 ascending colon fell over time on placebo, significantly so in the repeated doses study, possibly reflecting continuing water absorption in the absence of further input from the ileum. This fall was prevented by 3 doses of bisacodyl most likely due to the increased fluid observed in the small bowel but also possibly due to colonic secretion. While a single dose did not alter small bowel water content, repeated doses did. While the observed increase in colon water content was expected in line with previous in vitro and in vivo studies, the increase in the small bowel water content has not been previously reported. Bisacodyl's enteric coating is pH dependent, designed to start dissolving once the pill has left the acid environment of the stomach. Thereafter, dissolution is time dependent, with tablet break‐up and absorption of its metabolite BHPM (bis‐[p‐hydroxyphenyl]‐pyridyl‐2‐methane) occurring with a lag time of 4 hours^24^ by which time the tablet should have reached the distal ileum/proximal colon. Previous in vitro and in vivo studies both in animals and humans have demonstrated that direct administration of uncoated bisacodyl in the small bowel increase secretion. It is therefore possible that if transit is slow tablets may break up in the distal ileum releasing active drug thereby causing secretion. This event is probably more likely in constipated participants and if multiple doses are taken. The similar increase of small bowel motility immediately after both bisacodyl and placebo ingestion might reflect the disruption of fasting motility which is seen after fluid ingestion of even modest calorie intake.^19^ This could have been caused by the glass of water given to aid swallowing or the effect of the mechanical stimulus provided by the pill rather than a drug effect.

The current study shows that both single and repeated doses of bisacodyl accelerate whole gut transit and reduce time to defecation. This result is in line with a previous study conducted with scintigraphy in healthy subjects,^2^ but it is the first to observe this in constipated participants.

Multiple doses of bisacodyl also significantly increased the number of epochs when the colon volume change exceeds 20%, which are equivalent to the “mass peristalsis” as previously noted using cinefluorography after the laxative, oxyphenisatin.^13^ These changes in colon volume are likely to represent changes in long‐lasting tone which are well captured by MRI scans at 75‐minutes intervals. This is in keeping with previous studies demonstrating that bisacodyl suppositories induce a long‐lasting increase in rectal tone.^25^ Moreover, in vitro, bisacodyl increases the muscle tone of both longitudinal and circular human smooth muscle.^6^ Re‐analysis of previous studies performed in Nottingham show that such large colon volume changes occur 2.8 (0.8) times in 5 hours (0.56/hour) in the ascending colon after the highly effective osmotic laxative, Moviprep.^26^ The current repeated dose study found a similar rate 5.6 (2.2) in 7.5 hours (0.74/hour) after bisacodyl, in keeping with both being potent laxatives. Previous studies have shown that bisacodyl stimulates HAPCs 5–6 hours after oral administration so these colonic volume changes observed from 300 to 450 minutes after dosing are likely to be associated with movement of colonic contents driven by the propulsive HAPCs.^11^

Interestingly, while both single and repeated doses accelerated whole gut transit and reduced time to first defecation, only multiple doses increased the frequency of bowel movements and reduced stool consistency, as well as a number of epochs of mass peristalsis. Previous studies have suggested that the duration and extent of contact of oxyphenisatin with the colon wall determine the degree of response in terms of the number of mass peristalsis movements observed in healthy subjects.^13^ Whether this is the reason for our different results with a single and repeated dosages of bisacodyl remains to be elucidated. Alternatively, this may reflect the increased wateriness of chyme, possibly due to a cumulative effect of repeated doses.^13^

The strength of this study relates to the unique insights made possible by the non‐invasive, highly participant‐acceptable MRI, allowing simultaneous assessment of both secretion and motility, a feature which could be of value in evaluating other novel laxatives. Limitations to this study include the relatively small number of subjects studied, technical difficulties with some subjects in using wall movements to assess colonic motility, and the fact the study only included constipated participants with occasional use of laxatives. However, the mean whole gut transit time of our patients 63 hours is actually greater than the average transit time of 58 hours in constipated patients reported in a recent meta‐analysis^27^ so our results are likely to be relevant to most patients seen in the clinic. Furthermore, we were able to develop a new metric, the number of epochs with > 20% change in volume, which seems useful to measure mass peristalsis in a non‐invasive way. Ideally, we would have liked all participants to take part in both studies but practical reasons like inability to schedule visits and the complexities of COVID restrictions meant this was only possible for 6. Bowel habits may be altered during menstruation^28^ so we avoided scanning during menstruation but did not otherwise synchronize scanning with the menstrual cycle.

This study demonstrates that single‐dose bisacodyl 5 mg will be efficacious in “normalizing” the whole gut transit time and reducing the time to first defecation without altering stool consistency. When the dosage is repeated thrice there is also an increase in small bowel and colon water content, as well as increased stool frequency, reduced consistency, and shortened time to first defecation. Therefore, clinicians and patients could be advised that repeated 5 mg doses are likely to soften stools and would be well worth trying rather than increasing the dose to 10 mg which could be less well‐tolerated in some patients.

Future studies using MRI could throw light on the mode of action of a range of novel prokinetics and secretagogues currently being developed to treat constipation.^29^

## FUNDING

Research grant to the University of Nottingham from Sanofi. Sanofi was involved in the study design, preparation of the manuscript, and decision to publish.

## CONFLICTS OF INTEREST

RS has received research grants from Sanofi, Zespri, and Nestle and is a consultant for EnteroBiotix. MC is a consultant for Arena, Biocodex, PROMEDCS, Takeda, Nestle, RB, Mayoly. AA, RL, and BBDF are employees of Sanofi. All other authors declared no competing interests in this work.

## AUTHOR CONTRIBUTION

AA, ND, CH, CC, AA, RL, BB, MC, and RS wrote the manuscript; AA, ND, CH, CC, PG, AA, RL, BB, MC, LM, and RS designed the research. AA, ND, CH, and HW performed the research. AA, ND, CH, EG, SS, CC, AA, RL, BB, and RS analyzed the data.

## ETHICS STATEMENT

University of Nottingham Medical School Ethics Committee (Reference No: 420‐1911 and 419‐1911).

## DISCLAIMER

The views expressed are those of the author(s) and not necessarily those of the NHS, the NIHR, or the Department of Health and Social Care.

## Supporting information


Data S1.

## Data Availability

De‐identified individual participant data that underlie the reported results will be made available 3 months after publication for a period of 5 years after the publication date Proposals for access should be sent to robin.spiller@nottingham.ac.uk. The study protocol is included as a data supplement available with the online version of this article.
