# Supplementary material for: Magnetic Resonance Imaging Reveals Novel Insights into the Dual Mode of Action of Bisacodyl: A Randomized, Placebo‐controlled Trial in Constipation
Source: Clin Pharmacol Ther. 2024 Dec 16;117(5):1284–91. doi: 10.1002/cpt.3532 (PMC11993282; doi:10.1002/cpt.3532)
Supplement: Supplementary file 1 — Data S1. [file CPT-117-1284-s001.zip › cpt3532-sup-0009-Supinfo.docx]

# Supplement

## Inclusion Criteria

- Adult subjects meeting Rome IV criteria for Functional Constipation and self-medicating with occasional laxative no more than 4 times in the month before eligibility confirmation
- ≥18 years of age inclusive, at the time of signing the informed consent.
- Screening diary confirms they experience at least 2 days in the 7-day diary with either hard stools (Bristol Stool form Score 1 or 2) or no bowel movement
- BMI < 35 kg/m2
- Female subjects of childbearing potential must have a negative urine pregnancy test at screening. Subjects of childbearing potential or with partners of child bearing potential must agree to use methods of medically acceptable forms of contraception during the study and for 90 days after completion of study drug, (e.g. implants, injectable, combined oral contraceptives, barrier methods, true abstinence (when this is in line with the preferred and usual lifestyle of the patient) or vasectomised partners).
- Capable of giving signed informed consent.

## Exclusion Criteria

Subjects will be excluded from taking part in the study if any of the following criteria apply:

**Medical conditions:**

- Known hypersensitivity or other contraindications to bisacodyl use: such as ileus, intestinal obstruction, acute abdominal conditions including appendicitis, acute inflammatory bowel diseases, and severe abdominal pain associated with nausea and vomiting; and severe dehydration or rare hereditary conditions that may be incompatible with an excipient of the product (galactose intolerance, e.g. galactosaemia, fructose intolerance.
- Pregnancy and/or breast breast-feeding or positive urine pregnancy test History of known gastrointestinal organic disease (i.e. celiac disease, inflammatory bowel diseases).
- A positive diagnosis of irritable bowel syndrome based on the Rome IV criteria questionnaire which requires the patient to experience abdominal pain at least weekly.
- Reported history of previous major abdominal surgery (excluding appendectomy and cholecystectomy)
- Any medical condition making participation potentially compromising the participation in the study (e.g. diabetes mellitus, respiratory disease limiting ability to lie in the scanner, known allergy to one of the food products) as per investigator’s judgment.
- Contraindications for MRI scanning i.e. metallic implants, pacemakers, history of metallic foreign body in eye(s) and penetrating eye injury.
- Any condition incompatible with the patient’s participation to the study as per investigator’s judgment.
- Not willing or able agree to dietary restrictions required in 24 hours before each MRI study day.
- Anticipate need for antibiotics intake during the study.
- Unwilling to stop probiotics for the duration of the study period.
- Unable/ not willing to stop drugs known to alter GI motility including, regular loperamide, antispasmodics, (e.g. Buscopan®, mebeverine, peppermint oil, alverine citrate), eluxadoline opiates, monoamine oxidase inhibitors, phenothiazines, benzodiazepines, calcium channel antagonists for the duration of the study (Selective serotonin reuptake inhibitors and low dose tricyclic antidepressants (< 30mg amitriptyline or 50mg nortriptyline) will be recorded but provided dose is stable, will not be an exclusion criteria).
- Having taken part in a research study within 3 months

## Study design

These were randomized, investigator and patient blinded, placebo controlled, cross-over studies with 2 weeks wash out periods after which the ongoing presence of constipation was confirmed before proceeding. A daily stool diary was completed throughout the study including the washout period. Participants were contacted by phone to start the one-week eligibility confirmation period to ensure they still fulfilled the criteria for constipation symptoms prior to entering the second period of dosing. No subject failed to return to baseline after the 2-week washout. Subjects were asked to avoid apples, pulses, beans , peas, sweetcorn and spicier food that they were used to from the day before scanning. They were also asked to avoid alcohol, sports drinks and strenuous exercise the day before scanning

## Patient flow

We screened 34 participants of whom 5 were ineligible, 14 were randomized to the single dose and 15 to the repeated dose study. 2 in the single dose and 4 in the repeated dose study only completed period 1. In the repeated dose study 4 of the 15 randomized were excluded due to :1 claustrophobia, 1 study drug out of date, 2 took prohibited drug (opioid=1, antibiotics=1). Of the 12 receiving treatment in the single dose study, all completed the study but 1 was finally excluded from analysis when image analysis indicated a medical abnormality, later diagnosed as gastroparesis. Consort diagram (Figure 2). No one dropped out because of adverse events. Thus, the mITT and PP analysis was 11 in both studies. We did not synchronize scans with the menstrual cycle but did avoid scanning subjects during menstruation.

Figure S1 Consort diagram of participant flow from enrolment to analysis in both single and repeated dose studies

## Patient demographics

| Single dose study  Demographic Median (IQR) |  |
| --- | --- |
| N | 11 |
| Age (years) | 53 (23,69) |
| Gender = Female /Male | 9/2 |
| BMI | 25.5(23.4,29.0) |
| BM frequency/week | 4.7(2.6, 5.9) |
| BSFS | 2.0(1.5, 3.4) |

| Repeated dose study  Demographic median (IQR) |  |
| --- | --- |
| N | 11 |
| Age (years) | 54.5 (50.0, 59.8) |
| Gender = Male | 0 |
| BMI | 28.0 (23.3, 32.3) |
| BM frequency/week | 4.9 (5.2) |
| BSFS | 1.7 (2.5) |
|  |  |

Stool frequency and stool form score were obtained during screening prior to drug administration.

## Treatment supply and randomisation

Treatments were provided in numbered containers, active and placebo being identical in appearance. Sanofi prepared identical appearing active and placebo tablets sealed in numbered blister packs which were dispensed by Nottingham Pharmacy. However the covid pandemic meant the drug expired and Nottingham Pharmacy prepared a second batch using over-encapsulation with gelatin capsules so both active and placebo were identical. The randomization code was held in pharmacy and only broken once all images had been analyzed and all data queries were resolved before data lock occurred.

## MRI details

### T1 significance

T1 is a time constant describing the speed at which nuclear magnetization returns to its equilibrium distribution after being perturbed by a radiofrequency pulse by exchanging energy with its surroundings, and largely depends on water mobility. Liquid water has a long T1 at 3-4 seconds, but most tissues have a shorter T1 (e.g. fat 380 ms, liver 810 ms). Ascending colon T1 is increased by both psyllium and kiwifruit ^1 2^ reflecting the water trapping they induce. The T1 of the descending colon contents correlates with the water content of the next stool passed after the scan ^1^.

In this work the longitudinal relaxation time T1 was measured in the ascending colon and separately in the descending colon, using a single slice inversion recovery bTFE sequence with a preparatory 180° inversion pulse applied before acquiring the imaging data using a bTFE readout. The ROI size used for the T1 measurements were generally quite small, this reduced the likelihood of including colonic haustra. There was no absolute minimum size, however the software used to draw the regions did not allow for extremely small ROIs which would generate more noisy data. We have conducted an experiment that looked at the influence of the size of the ROIs and found very little difference between the results from different ROI sizes ^3^ Figure 2 shows an example of T1 measurement from this study.

### Figure S2 T1 images

### Imaging details

Imaging was carried out on a state-of-the-art, research dedicated 3.0T Scanner with a parallel imaging SENSE 16-element abdominal body coil wrapped around the torso. A range of MRI sequences were used including:

1. A high resolution balanced turbo field echo sequence (bTFE ) to acquire images of the contents of the ascending and descending colon (TR = 2.5 ms, TE = 1.27 ms, acquired resolution = 1.50 mm x 1.50 mm, reconstructed resolution= 0.86 x 0.86, 8 slices 7 mm thick, 0.58 mm gap, flip angle 45°, NEX = 4) ^4^.

2. The longitudinal relaxation time T1 was measured in the ascending colon and separately in the descending colon, using a single slice inversion recovery bTFE sequence with a preparatory 180° inversion pulse applied before acquiring the imaging data. The slice for the T1 was chosen from the high-resolution images and was chosen as the slice which provides the best cross-section through the colonic segment maximising the colonic content to sample. The parameters for this sequence were a field of view of 400 (HF) × 280 (AP) mm; 7 mm slice thickness; half Fourier acquisition; 256 × 256 matrix; repetition time/echo time 2.72/1.36 milliseconds; flip angle 45°. Data was acquired from eight different inversion times (TI; time between inversion pulse and imaging pulses) ranging from 0.1 to 5 seconds as previously described^2^. A 10 second gap between each acquisition to allow the system to return to equilibrium.

3. Colonic images were analysed by manually outlining the colon to create regions of interest (ROI) from images acquired using a 2-station 3D coronal dual echo fast field echo sequence with mDIXON image reconstruction ^20,21^Volumes were calculated by summing the area of the ROI X slice thickness, as previously described ^4^.

3. A single shot, fast spin echo sequence (rapid acquisition with relaxation enhancement, RARE) to acquire in a single breath-hold 20 coronal images with in-plane resolution interpolated to 0.78 mm x 0.78 mm and a slice thickness of 7 mm, with no gap between slices (TR = 8000 ms, TE = 400 ms, SENSE 2.0). This sequence yields high intensity signals from areas with fluid and little signal from body tissues and is used to measure bowel water content.

4. Small bowel Motility was assessed at baseline and postprandially using a cine MRI bTFE acquisition. Scanning was acquired over multiple slices during gentle free breathing with temporal resolution of 1s per image set. 2 slices were acquired during each 60 s acquisition with a total of 6 coronal slices across the small bowel. Analysis was carried out following image registration as previously described ^5^ ^6^. This quantified the motility of the bowel using the pixel signal changes through the time series, within a defined region of interest, placed over the visible small bowel following image registration to remove diaphragmatic movements ^5^.

5. Colonic Motility was assessed at baseline and every 75 minutes for 7.5 hours post treatment using a multi-slice cine MRI bTFE acquisition with 2 slices, 1 positioned over the AC and 1 over the DC with analysis as previously published ^6^. Temporal resolution was 1 second and data was acquired over a 10-minute time period of gentle free breathing. The numbers of data sets for AC motility were markedly reduced because of technical difficulties. These related to suboptimal slice alignment and colonic appearance (with the haustra more obvious compared to previous studies with Movicol® when the bowel was fuller and more homogeneous^7^) making it difficult to distinguish genuine colonic movement from respiratory artefact. Sporadic large scale mass movements were also not well tracked by the software which relies on changes from image to image being small. This type of motion has not been tested before using this methodology, so this was an unexpected finding.

6. The position of the transit pills and the colonic volumes were assessed using a 2-station 3D coronal dual echo fast field echo sequence with mDIXON image reconstruction (TE = 1.1/2.1ms TR = 3.3 ms SENSE factor = 2, reconstructed in-plane resolution 0.98 x 0.98 mm, acquired slice thickness 3.6 mm, interpolated to 1.8 mm with a reconstruction matrix of 432). These two sets of images were combined into a single image of matrix size 543 (HF) × 432 (LR) and 37 slices of thickness 5.4 mm with data acquired during 2 expiration breath hold of 20 s (1 for each station). Transit was assessed using the scoring system previously described ^8^. Each pill was scored based on which colonic segment it lay in, scores ranging from 6 for the lower ascending colon to 1 for the rectum and 0 if expelled. The transit time in hours was calculated from a validated linear equation linking average weighted position score as previously published ^8^

7. Most image sets were acquired on an expiration breath-hold with duration between 13 and 24 seconds depending on the sequence, monitored using a respiratory belt. Including set-up and imaging the volunteers spent approximately 35 minutes inside the magnet for every time point, and the rest of the time sitting upright in an adjacent room.

## Figures

### Figure S3 Small bowel motility

### Figure S4 AC volumes changes over time before and after intake of study drug.

### **Figure S5 TC volume changes over time before and after intake of study**

### Figure S6 DC volume over time before and after intake of study drug (A. single and B. repeated doses)

### Figure S7 RSC volume change over time before and after intake of study drug

## Video Legend

Sagittal sections showing Ascending colon (left) and Descending colon (right). These MRI Images were acquired every second and the video is speeded up by x10 to show 10 minutes of acquisition in 1 minute of video. Early in the sequence, around 8-11 secs the ascending colon shows strong contractions and propulsion of a large bolus upwards towards the hepatic flexure followed at around 30 secs by the appearance of a large bolus of content in the descending colon, a second bolus is seen arriving in the descending colon at around 46 seconds.

1. Major G, Murray K, Singh G, et al. Demonstration of differences in colonic volumes, transit, chyme consistency, and response to psyllium between healthy and constipated subjects using magnetic resonance imaging. *Neurogastroenterol Motil* 2018;30(9):e13400. doi: 10.1111/nmo.13400 [doi]

2. Wilkinson-Smith V, Dellschaft N, Ansell J, et al. Mechanisms underlying effects of kiwifruit on intestinal function shown by MRI in healthy volunteers. *Aliment Pharmacol Ther* 2019;49(6):759-68. doi: 10.1111/apt.15127 [doi]

3. Omar NF. Diffusion weighted imaging and relaxometry in abdominal organs. University of Nottingham, 2016.

4. Pritchard SE, Marciani L, Garsed KC, et al. Fasting and postprandial volumes of the undisturbed colon: normal values and changes in diarrhea-predominant irritable bowel syndrome measured using serial MRI. *Neurogastroenterol Motil* 2014;26(1):124-30. doi: 10.1111/nmo.12243 [doi]

5. Khalaf A, Hoad CL, Menys A, et al. MRI assessment of the postprandial gastrointestinal motility and peptide response in healthy humans. *Neurogastroenterol Motil* 2018;30(1) doi: 10.1111/nmo.13182 [published Online First: 2017/09/01]

6. Menys A, Hamy V, Makanyanga J, et al. Dual registration of abdominal motion for motility assessment in free-breathing data sets acquired using dynamic MRI. *Phys Med Biol* 2014;59(16):4603-19. doi: 10.1088/0031-9155/59/16/4603 [doi]

7. Menys A, Hoad C, Spiller R, et al. Spatio-temporal motility MRI analysis of the stomach and colon. *Neurogastroenterol Motil* 2019;31(5):e13557. doi: 10.1111/nmo.13557 [doi]

8. Chaddock G, Lam C, Hoad CL, et al. Novel MRI tests of orocecal transit time and whole gut transit time: studies in normal subjects. *Neurogastroenterol Motil* 2014;26(2):205-14. doi: 10.1111/nmo.12249 [doi]

Legend to Figures

1.7.1 Figure S1 Consort diagram of participant flow from enrolment to analysis in both single and repeated dose studies

1.7.2

Figure S2 showing T1 images and data from Day 3 at 275 mins after taking 3 repeated 5mg dose of bisacodyl. Inversion time (TI) of each of the images are shown with the 0.1 s data shortly after the AC is perturbed by the MRI radiofrequency pulse. 1.0 s data is an intermediary time and 5.0 s data the longest one acquired in this study where shorter T1 tissues will have realigned with the magnetic field. Panel A: Sagittal T1 images of AC for Bisacodyl arm. Measured T1 was 1.9s for green ROI shown. Panel B: T1 images for placebo arm of study. Measured T1 was 0.3 s for green ROI shown. Panel C: Normalised graph showing all TI data. Data normalised to TI = 5.0 s. Bisacodyl data with longer T1 (more watery colonic content) takes longer to return to equilibrium value.

1.7.3

Figure S3 Small bowel motility

*Changes in small bowel motility in* arbitrary units *over time before and after intake of study drug .* Data shown as *Mean (SD). SB motility rose promptly after ingesting both bisacodyl and placebo and remained elevated until 450 minutes. By the next day it had returned to baseline. There were no significant differences between bisacodyl and placebo. 2-way Repeated Measures ANOVA showed a significant effect of time p<0.001 but no effect of treatment, p=0.78. Post hoc comparisons showed motility at time 75 - 450 minutes was significantly greater than time 0, all comparisons p=<0.005 .*

1.7.4

Figure S4 AC volumes changes over time before and after intake of study drug. Data shown as mean(SD)

*Single dose study showed no significant change while after repeated doses there was a significant fall from 150-450 minutes, 0=0.03.*

1.7.5

Figure S5 DC volume over time before and after intake of study drug (A. single and B. repeated doses*) Data shown as mean(SD) There was no significant difference between bisacodyl and placebo.*

1.7.6

Figure S6 RSC volume change over time before and after intake of study drug. *Data shown as mean(SD) There was a significant increase in SRC volume after a single dose, p=0.03 but this was not significant in the repeated dose study, p=0.22*.
