# Supplementary figures and images for: Magnetic Resonance Imaging Reveals Novel Insights into the Dual Mode of Action of Bisacodyl: A Randomized, Placebo‐controlled Trial in Constipation
Source: Clin Pharmacol Ther. 2024 Dec 16;117(5):1284–91. doi: 10.1002/cpt.3532 (PMC11993282; doi:10.1002/cpt.3532)

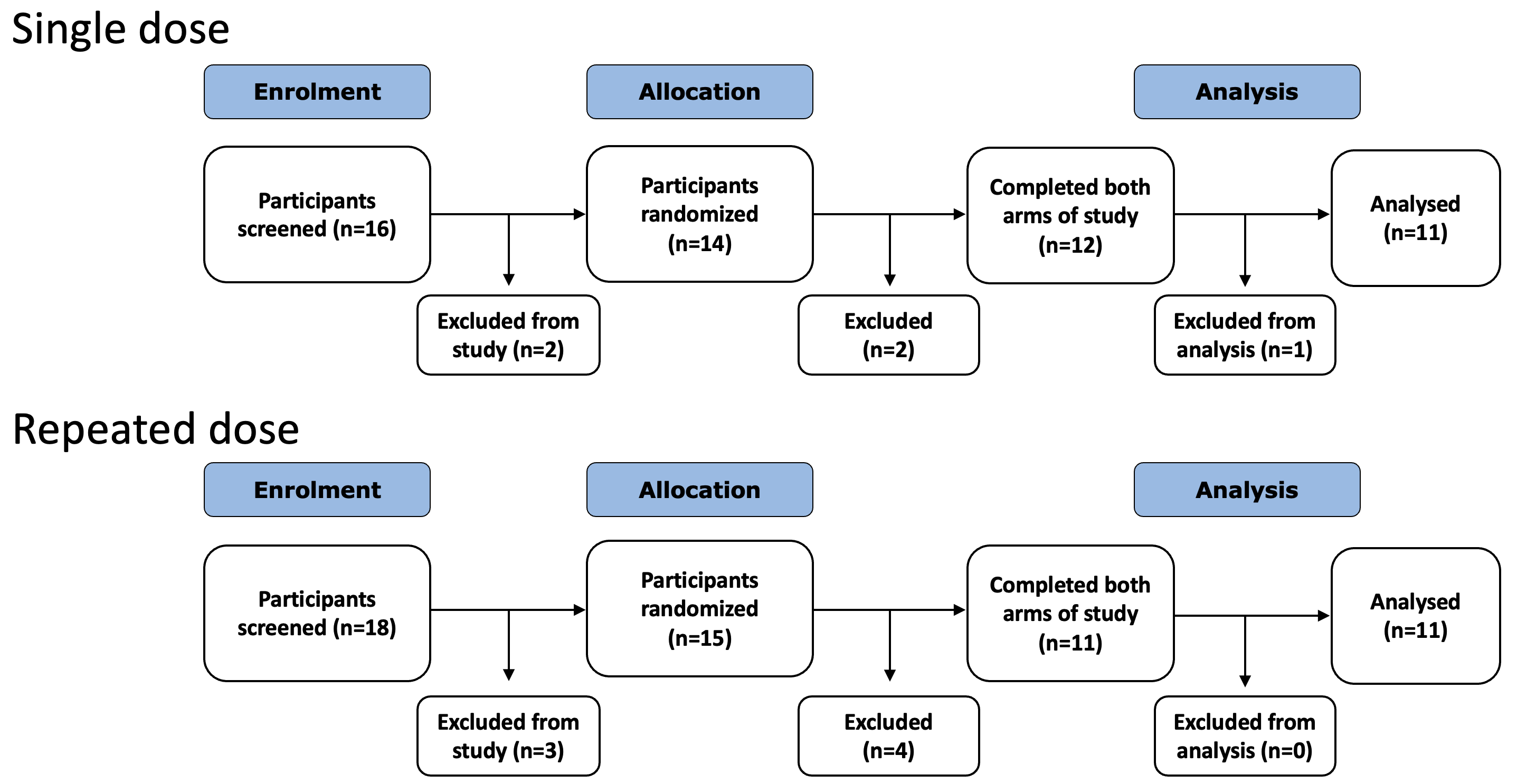

Supplement: Supplementary file 1 — Data S1. [file CPT-117-1284-s001.zip › cpt3532-sup-0001-FigureS1.tif]

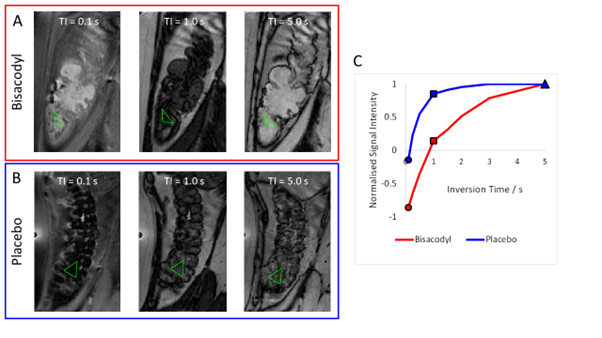

Supplement: Supplementary file 1 — Data S1. [file CPT-117-1284-s001.zip › cpt3532-sup-0002-FigureS2.tif]

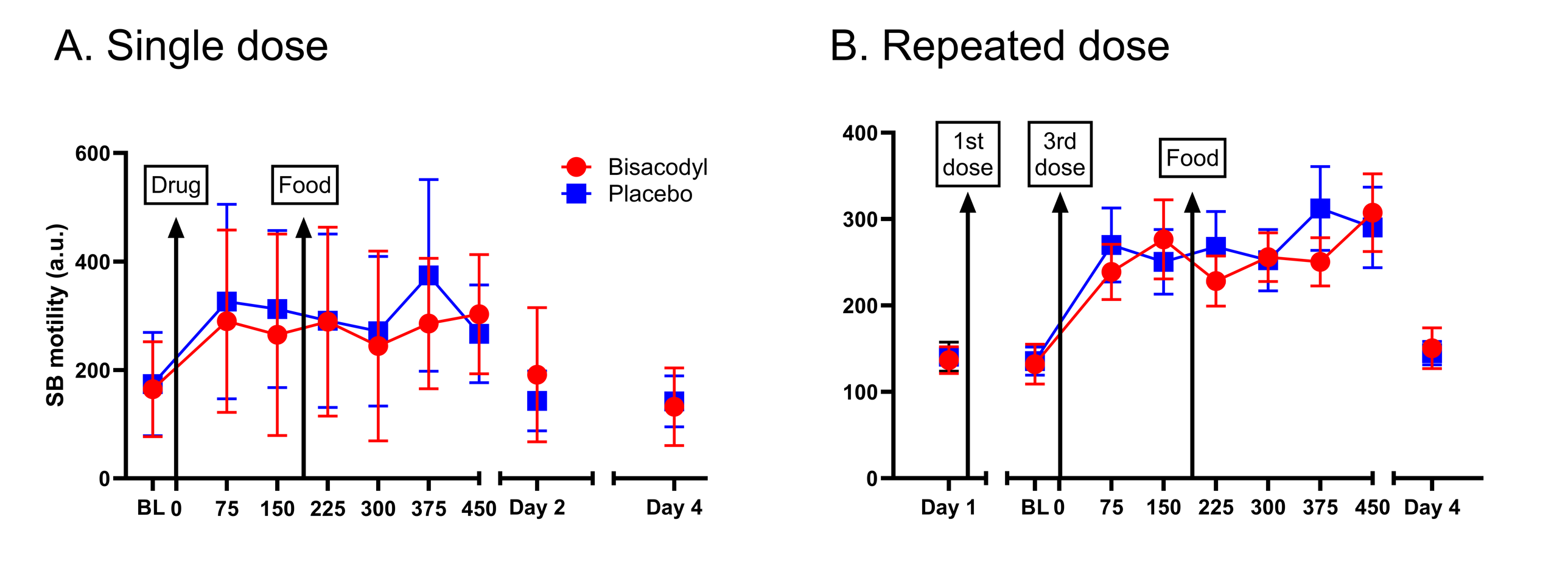

Supplement: Supplementary file 1 — Data S1. [file CPT-117-1284-s001.zip › cpt3532-sup-0003-FigureS3.tif]

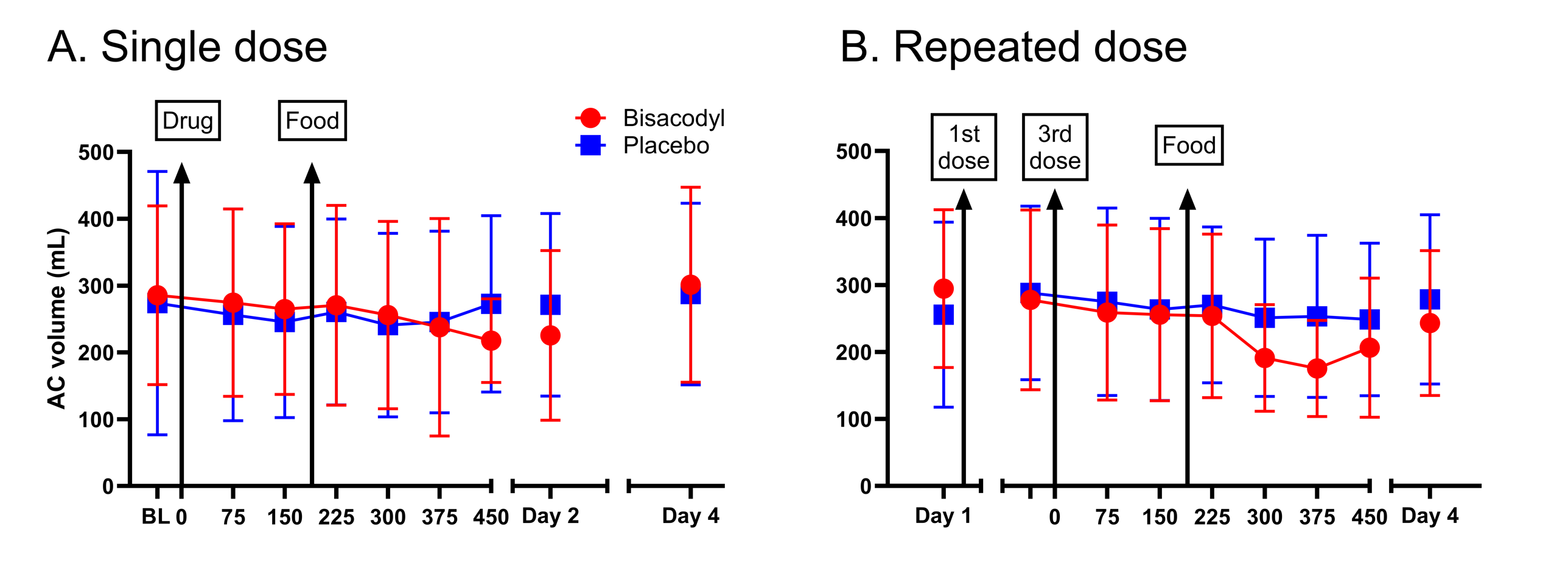

Supplement: Supplementary file 1 — Data S1. [file CPT-117-1284-s001.zip › cpt3532-sup-0004-FigureS4.tif]

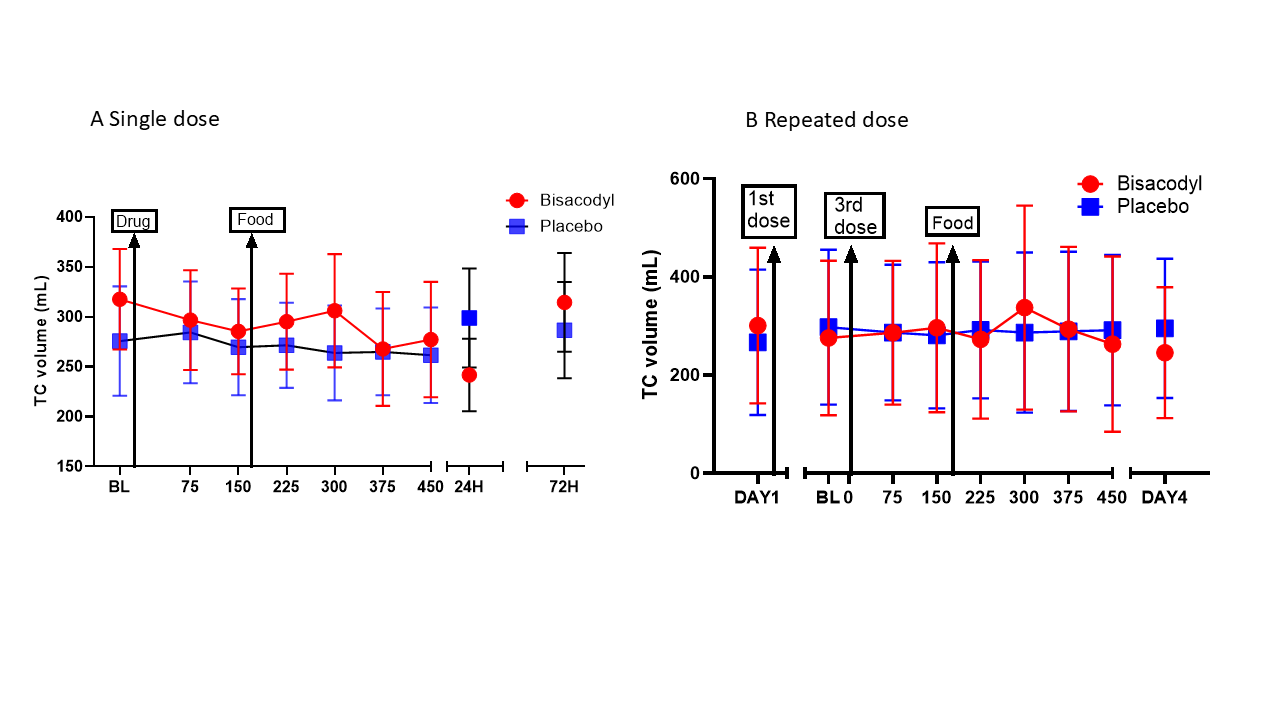

Supplement: Supplementary file 1 — Data S1. [file CPT-117-1284-s001.zip › cpt3532-sup-0005-FigureS5.tif]

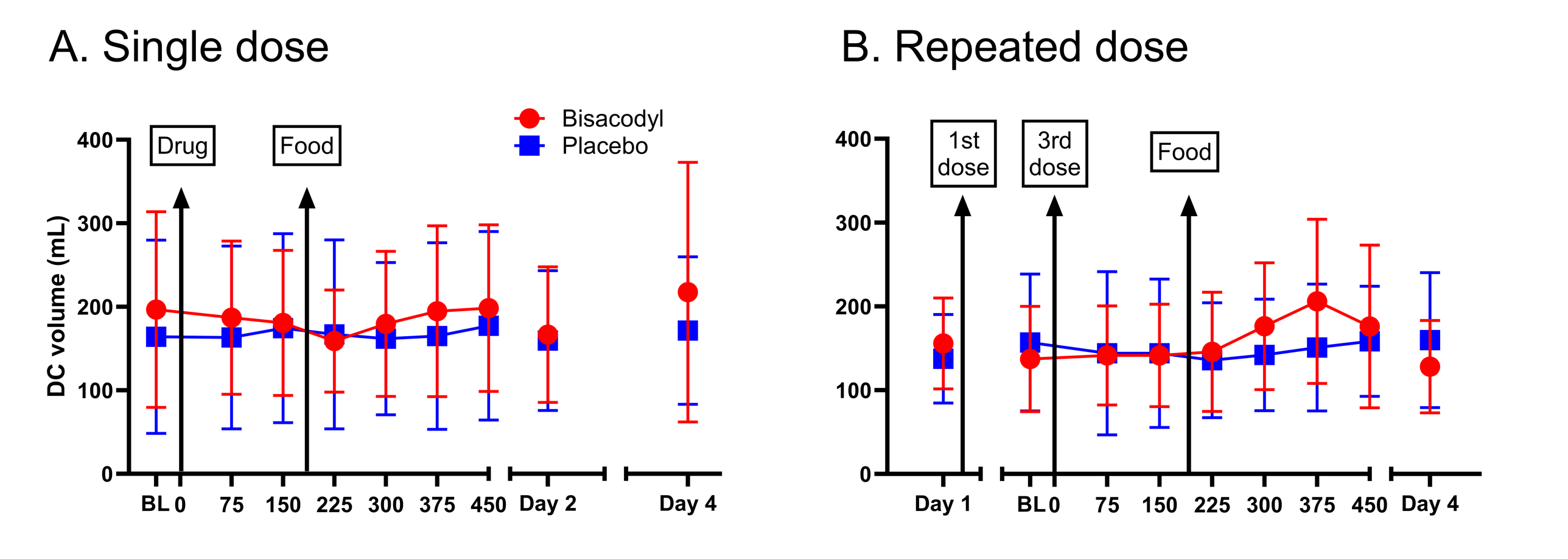

Supplement: Supplementary file 1 — Data S1. [file CPT-117-1284-s001.zip › cpt3532-sup-0006-FigureS6.tif]

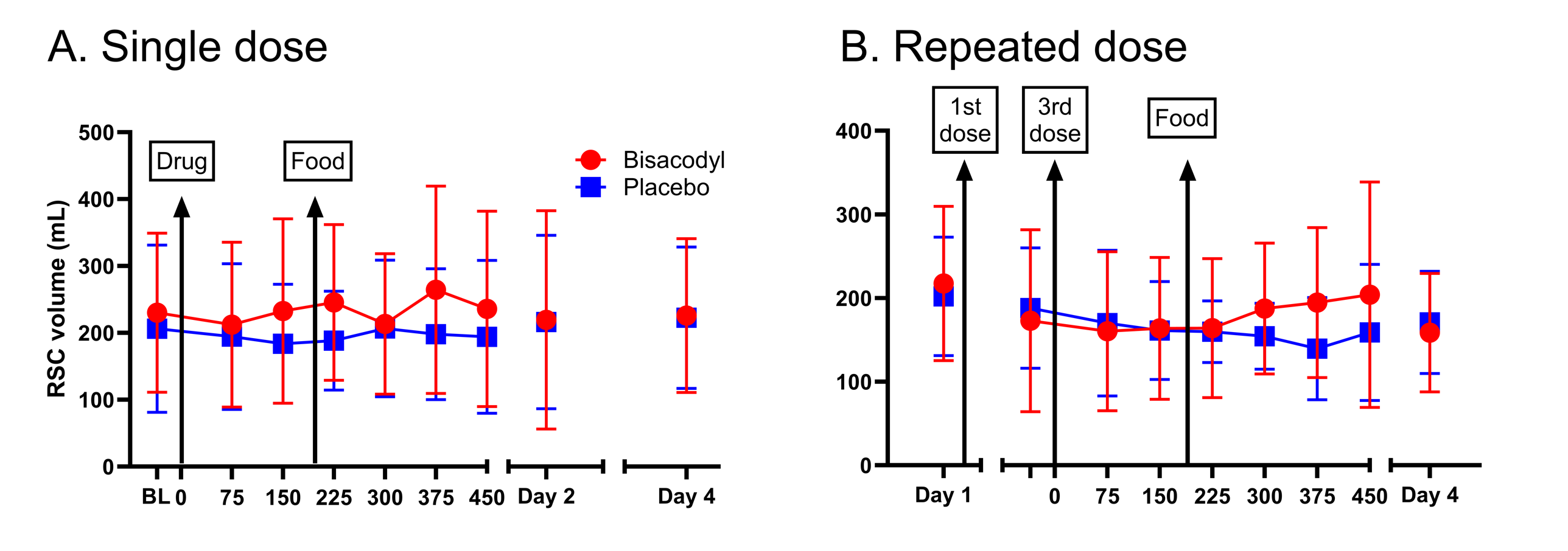

Supplement: Supplementary file 1 — Data S1. [file CPT-117-1284-s001.zip › cpt3532-sup-0007-FigureS7.tif]
